# The APOLLO trial: a proof-of-concept study for vitamin A nasal drops in COVID-19-related postinfectious olfactory dysfunction

**DOI:** 10.1093/chemse/bjag001

**Published:** 2026-01-20

**Authors:** Zhu Hui Yeap, Rashed Sobhan, Sara L Bengtsson, Saber Sami, Allan B Clark, Ramesh Vishwakarma, James Boardman, Juliet High, Gabija Klyvyte, Mehmet Ergisi, Thomas Hummel, Carl M Philpott

**Affiliations:** Rhinology & Olfactology Research Group, Norwich Medical School, University of East Anglia, Norwich, United Kingdom; Norfolk Smell & Taste Clinic, James Paget University Hospital NHS Trust, Gorleston, United Kingdom; Sir Peter Mansfield Imaging Centre, University of Nottingham, Nottingham, United Kingdom; Norwich Medical School, University of East Anglia, Norwich, United Kingdom; School of Psychology, University of East Anglia, Norwich, United Kingdom; Norwich Medical School, University of East Anglia, Norwich, United Kingdom; Norwich Medical School, University of East Anglia, Norwich, United Kingdom; Norwich Medical School, University of East Anglia, Norwich, United Kingdom; SmellTaste (Registered Charity), Bicester, United Kingdom; Norwich Medical School, University of East Anglia, Norwich, United Kingdom; Norwich Medical School, University of East Anglia, Norwich, United Kingdom; Norwich Medical School, University of East Anglia, Norwich, United Kingdom; Interdisciplinary Center “Smell and Taste”, Department of Otorhinolaryngology, Technical University of Dresden, Dresden, Germany; Rhinology & Olfactology Research Group, Norwich Medical School, University of East Anglia, Norwich, United Kingdom; Norfolk Smell & Taste Clinic, James Paget University Hospital NHS Trust, Gorleston, United Kingdom; SmellTaste (Registered Charity), Bicester, United Kingdom

**Keywords:** intranasal vitamin A, olfactory dysfunction, postinfectious olfactory dysfunction, COVID-19, post-COVID smell disorder, MRI of cerebral olfactory units

## Abstract

Postinfectious olfactory dysfunction (PIOD) is common in COVID-19 patients. This 2-arm double-blinded randomized controlled trial (RCT) aimed to establish proof-of-concept for vitamin A versus placebo as a treatment modality for patients with PIOD. This study compared 9,000 IU daily self-administered vitamin A intranasal drops versus peanut oil drops over 12 wk in COVID-19 patients with PIOD. Outcome measures included: olfactory bulb volume (OBV), olfactory sulcus depth, cerebral functional MRI blood oxygen level dependent (BOLD) signal, Sniffin’ Sticks TDI score, SSParoT, olfactory disorder questionnaire (ODQ) score, and brain-derived neurotropic factor (BDNF) levels were collected from participants at baseline and after trial intervention at 12 wk. Fifty-seven PIOD were recruited in the trial and allocated to vitamin A or placebo arm at a 2:1 ratio. After withdrawals and exclusions, 30 participants in the vitamin A arm and 15 in the placebo arm were analyzed. There was no significant difference in the change in OBV between both groups. Aside from an improvement in the quality-of-life component of ODQ questionnaire scores (*P* = 0.01), there were no significant differences in any of the other secondary outcome measures. This proof-of-concept trial has demonstrated no significant effect of intranasal vitamin A on olfactory function in COVID-19 PIOD patients. Further work is required to identify other therapeutic agents in the management of PIOD or evaluate a different PIOD cohort with non-COVID etiology.

## 1. Introduction

### 1.1 Background

Loss of smell is a common complaint in adults, with the main diagnostic groups being sinonasal disease (62%) and postinfectious olfactory loss (PIOD) (19%) (Hummel et al. 2017a; Damm et al. 2019). Following the SARS-CoV-2 pandemic, there has been a new cohort of patients with PIOD. Up to 60% of those contracting the virus are affected by this symptom (Menni et al. 2020; Parma et al. 2020). The majority recover their sense of smell within 4 wk, but current data suggest that 7% to 18% have continued smell loss and do not recover spontaneously (Boscolo-Rizzo et al. 2024; Lötsch et al. 2024; Saccardo et al. 2025); with estimates of up to 1 million people in the United Kingdom having PIOD due to COVID-19 (Gokani et al. 2022; Lechner et al. 2022). For those affected, there are major impacts on their safety (e.g. gas safety), self-esteem, and quality of life, especially when parosmia (i.e. a distorted sense of smell) is present as well (Bonfils et al. 2005; Philpott and Boak 2014; Gopinath et al. 2016; Erskine and Philpott 2020; Philpott et al. 2021).

The SARS-CoV-2 virus infects respiratory tract cells via interaction with the ACE2 protein on the target cell surface, and cleavage of the S-protein by the protease TMPRSS2 enables this to occur. The ACE2 protein is commonly expressed in the respiratory tract and predisposes the cells to viral invasion (Brann et al. 2020). A leading theory on the key mechanism of action has been that the sustentacular cells, horizontal basal cells, and Bowman's gland cells in the olfactory epithelium express genes that code for both the TMPRSS2 protease and the ACE2 receptor, based on evidence produced by several research groups (Butowt and von Bartheld 2021). Viral infection of these cells leads to a disruption of the turnover and function of olfactory receptor neurons (ORNs), resulting in anosmia. Loss of ORN function can lead to neurodegeneration (Jafek et al. 2002) and volume reduction of the olfactory bulbs and orbital sulci (Seubert et al. 2013). Although the olfactory system can regenerate (Schwob 2002), this sometimes fails following viral injury to the receptors, and recent evidence from UK Biobank participants demonstrated the significant impact COVID-19 has had on the olfactory areas of the brain (Douaud et al. 2022), suggesting a degeneration through olfactory pathways or the loss of sensory input due to anosmia.

Vitamin A offers a potential treatment option for olfactory loss due to ORN damage. It is metabolized to retinoic acid (RAc), which, as a transcription regulator, is important in tissue development and regeneration (Balmer and Blomhoff 2002). When vitamin A is converted to retinoic acid, it is thought to control olfactory progenitor cell differentiation; therefore, it will prevent exhaustion of the stem cell supply or accumulation of nonfunctional immature neurons (that are not processing odors) (Paschaki et al. 2013). Therefore, it is theorized that topical vitamin A treatment will encourage regeneration of the olfactory epithelium, which is damaged by respiratory viruses responsible for the common cold, and help to restore the sense of smell in sufferers.

Apolipoprotein E (ApoE) is a crucial protein for lipid metabolism, and expression varies by genotype with 3 isoforms: E2, E3, and E4. The ApoE4 allele is one of the most well-known risk factors for Alzheimer's dementia (AD), with ApoE3 conferring an intermediate risk of AD, and ApoE2 being associated with neuroprotection (McLaren and Kawaja 2024). Homozygous carriers of 2 ApoE4 alleles have the greatest risk of earlier onset AD than heterozygous carriers (Ward et al. 2012). As degeneration of the olfactory bulb and subsequent olfactory dysfunction is one of the earliest pathologic signs of AD (Devanand 2016), the ApoE genotype may potentially influence the neuro-regenerative response to vitamin A as well.

We have previously conducted a systematic review, identifying 4 prior studies utilizing vitamin A as a treatment modality for olfactory loss (Duncan and Briggs 1962; Garrett-Laster et al. 1984; Reden et al. 2012; Addison and Philpott 2018). In particular, a pseudo-randomized clinical trial conducted in Germany in 2017 delivered intranasal vitamin A at a dose of 10,000 International Units (IU) per day for 8 wk. In 124 patients with PIOD, it demonstrated a minimum clinically important difference in olfactory function in 37% of those receiving vitamin A, compared to 23% receiving smell training alone (Hummel et al. 2017b). Although this study provided key information regarding the safety and efficacy of vitamin A for PIOD patients, it had unbalanced treatment groups and pseudo-randomization. As such, further rigorous scientific evidence is necessary to demonstrate proof of concept evidence for intranasal vitamin A.

### 1.2 Aims and objectives

The overarching aim of this study was to undertake a 2-arm RCT of intranasally delivered vitamin A versus placebo (peanut oil) to determine proof of concept. The following objectives were selected to provide physical evidence of the impact of vitamin A, and ApoE genotype, on:

1. Olfactory bulb and right orbital sulcus volume using MRI volumetric data.
2. White matter structural connectivity between these brain areas with diffusion MRI (dMRI).
3. Neural activation in the amygdala, temporal piriform, and insular cortices, as indicated by an average signal increase of 0.9 in the primary olfactory cortex using fMRI (Han et al. 2018).
4. Psychophysical smell test scores.
5. Quality of life.
6. Subgroup effect in outcomes between those with and without parosmia.
7. Pleasantness of 8 odors only in participants with parosmia.

## 2. Methods

### 2.1 Trial design

The study was conducted as a 2-arm randomized placebo-controlled double-blind trial comparing approximately 9,000 IU daily vitamin A self-administered intranasal drops versus matched peanut oil drops delivered over 12 wk in patients with PIOD. The trial was conducted in compliance with the approved protocol, the Declaration of Helsinki (2008), the principles of good clinical practice (GCP) as laid down by the Commission Directive 2005/28/EC with implementation in national legislation in the United Kingdom by Statutory Instrument 2004/1031 and subsequent amendments, the UK Data Protection Act, and the UK Policy Framework for Health and Social Care Research. The study was not considered to be a Clinical Trial of an Investigational Medicinal Product (CTIMP) by the Medicines & Healthcare products Regulatory Agency (MHRA), and as such did not need a clinical trials authorization as a proof-of-concept study. The study took place at the University of East Anglia Wellcome-Wolfson Brain Imaging Centre (UWWBIC).

### 2.2 Registration and ethics approval

The trial registration number is ISRCTN13142505 (https://doi.org/10.1186/ISRCTN13142505). The trial received NHS Research Ethics Committee (REC) approval from the West Midlands—Black Country REC, reference number 21/WM/0179, and Health Research Authority approval.

### 2.3 Participants

Written informed consent to enter and be randomized into the trial has been obtained from participants, after explanation of the aims, methods, benefits, and potential hazards of the trial and any trial-specific procedures. Participants were recruited from the tertiary referral Norfolk Smell & Taste Clinic located at the James Paget University Hospital and were screened for fulfillment of eligibility criteria outlined below.

#### 2.3.1 Participant inclusion criteria

A partial or total loss of smell due to post-viral olfactory loss as confirmed on history, examination, and with a smell test (TDI) score of < 31/48 and within 3 years of the precipitating viral infection.

#### 2.3.2 Participant exclusion criteria

1. Participants with a history of:
  - Chronic rhinosinusitis with/without nasal polyposis
  - Severe nasal septal deviation
  - Major prior head injury
  - Congenital olfactory loss
  - Use of concurrent intranasal medications or possible medications known to affect olfaction
  - Chronic renal disease
  - Chronic hepatic disease
  - Allergy to peanuts, soy, or vitamin A/E (drops contain peanut oil and small amounts of vitamin E)
2. Significant medical, surgical, or psychiatric disease that, in the opinion of the principal investigator, would have affected subject safety or influenced the study outcomes
3. Currently taking oral vitamin A supplements, anticoagulants, or tetracyclines
4. Age of less than 18 or over 65 years
5. Pregnant women and women of childbearing age not using an effective contraceptive
6. Participants unsuitable for MRI due to metal implants, such as pacemaker, etc., as is standard for MRI scanning, or who move excessively during scanning.
7. Evidence from endoscopy or the initial MRI scan of:
  - Participants with any endoscopic findings of:
    - Chronic rhinosinusitis with/without nasal polyposis
    - Severe nasal septal deviation (preventing passage of 4 mm endoscope)
    - Other inflammatory sinonasal disease
  - Participants with MRI changes indicating edema in the sinuses and/or olfactory clefts
8. Any participant with a combined OBV of > 90 mm^3^ was excluded as it is unlikely they would demonstrate a significant increase in overall volume based on previous studies of OBV (Rombaux et al. 2012); this was revised from 85 mm^3^ in our protocol to avoid additional exclusions.
9. Participation in another trial in the prior 4 mo.

Patients with allergic rhinitis were not excluded, but this information was also recorded as a baseline characteristic.

### 2.4 Interventions

Study participants self-administered vitamin A drops intranasally via dropper daily for 12 wk. Participants recruited between July and October 2022 received placebo or vitamin A drops (Vitadral Tropfen, Aristo Pharma GmbH, Berlin, Germany) at a dose of 8,133.3 IU once daily for 12 wk (2 drops per nostril). Due to the discontinuation of the manufacture of Vitadral Tropfen drops and current stocks reaching expiry date, the study was paused whilst an alternative vitamin A nasal drop product was sourced. The closest available match chosen was Coldastop after assessment to ensure its use would have no impact on the study outcome measures. The remaining participants recruited after the restart in 2024 received vitamin A drops (Coldastop Nasen-Öl, Desitin Arzneimittel GmbH, Hamburg, Germany) at a dose of 9,000 IU (3 drops per nostril twice daily) for 12 wk; 12 drops daily were required with the new product to achieve the same dose as given previously, as the strength of Coldastop was 8.25 mg/mL vitamin A compared to Vitadral Tropfen at 30.2 mg/mL. The placebo drop regimen was changed to match the revised requirement of 12 drops daily.

The dose and duration were based on the 2017 study by Hummel et al. (2017b). All participants were dispensed the drops once eligibility was confirmed. The participants of both arms were instructed on how to apply the drops using the Kaiteki position to ensure that the drops roll into the olfactory cleft (Mori et al. 2016).

### 2.5 Outcomes

#### 2.5.1 Primary outcomes

Primary outcome measure: Olfactory bulb volume (OBV) (on MRI scan)

#### 2.5.2 Secondary outcomes

- Right orbital sulcus depth (MRI scan)
- Blood oxygen level dependent (BOLD)—signal in primary olfactory areas: amygdala, piriform, and insula (fMRI scan)
- Psychophysical smell test (TDI) score
- SSParoT score in individuals with parosmia
- Olfactory disorders questionnaire (ODQ) score
- Brain-derived neurotrophic factor (BDNF) (pg/mL)
- Apolipoprotein E genotype

### 2.6 MRI scan OBV, sulcus measurements, and fMRI changes

For participants in both arms of the study, MRI data were taken at baseline and at 12 wk to measure change in the OBV, regional BOLD activation to measure changes during resting state and while passively smelling odors, as well as diffusion tensor imaging (DTI) data to measure changes in structural connectivity. During odor fMRI scanning, participants passively perceived phenethyl alcohol (rose-like pleasant smell) and hydrogen sulfide (rotten eggs—unpleasant), and odorless air in repeated, pseudo-randomized trials. Odors were delivered via an olfactometer (OL023, Burghart, Wedel, Germany). Each odor stimulus was delivered in a pre-randomized pattern for 5 s per trial, followed by 20 s of odorless air. The trials were divided into 3 sessions with 12 trials/session. The same scanning set-up was used at both visits.

#### 2.6.1 MRI scan parameters

Brain MRI data were acquired using a Siemens 3T Magnetom Prisma scanner with a 32-channel phased array receiving coil. At baseline and follow-up visits, we acquired a 3D structural T1-weighted Magnetization-Prepared Rapid Acquisition Gradient Echo image (MPRAGE) with repetition time (TR)/echo time (TE): 2,200/2.17 ms; flip angle (FA): 9°; field of view (FOV): 224 × 224 mm^2^; voxel size: 1 × 1 × 1 mm^3^; slice thickness: 1 mm; number of slices: 208. T2-weighted high-resolution images were acquired using TR/TE: 5,500/110 ms; FA: 150°; FOV: 256 × 256 mm^2^; voxel size: 0.47× 0.47 × 1.2 mm^3^ with no gap between slices.

For the odor fMRI scan, we used a gradient echo pulse sequence of 128 volumes/session; voxel dimensions (mm) [2.4 2.4 3.3]; TR/TE = 2,500/30 ms, FA = 90°, image size: [92 92 35]. A 10-min-long resting-state fMRI block consisting of single shot echo planar imaging (EPI) sequence was acquired with TR/TE: 1,500/22 ms; FA: 70°, interleaved 60 slices, GRAPPA 2; multiband acceleration factor: 2; FOV: 192 × 192 mm^2^, voxel size: 2.5 × 2.5 × 2.5 mm^3^, rendering a total of 354 volumes per participant. All participants were scanned with a head-first supine position and instructed to focus on a marked cross on the screen visible through a mirror placed on the head coil during the rsfMRI scan. A 24-min-long multi-shell diffusion MR was acquired with TR/TE: 3,230/89.20 ms; FA: 78°; FOV: 210 × 210 mm^2^, 1.5 mm^3^ isotropic voxel resolution, multiband acceleration factor: 4; b-values 0/1,500/3,000 s/mm^2^, 197 diffusion directions, each acquired twice with opposite phase encoding direction (anterior-posterior, AP and posterior-anterior, PA) to facilitate robust correction against distortions.

### 2.7 Psychophysical smell test, parosmia test, and olfactory disorders questionnaire

At baseline and follow-up visits, all participants underwent the Sniffin’ Sticks extended smell test to assess changes in olfactory performance through the threshold (T), discrimination (D), and identification (I) scores validated for a UK setting (Langstaff et al. 2021). The total smell test (TDI) was scored out of a maximum of 48. Participants were also asked about the presence of parosmia symptoms, and those with parosmia also underwent one additional assessment with the Sniffin’ Sticks parosmia test (SSParoT) (Liu et al. 2020). The olfactory disorders questionnaire assessed quality-of-life impact (Langstaff et al. 2019).

### 2.8 Brain-derived neurotrophic factor (BDNF)

Non-adherence to trial medication was assessed by bottle weight at 12 wk and through collecting blood samples to measure brain-derived neurotrophic factor (BDNF) as a proxy for a measure of the use of vitamin A.

### 2.9 Apolipoprotein E (ApoE)

At the follow-up visit, participants were consented separately for blood samples to be taken to check for ApoE gene carrier status. This was correlated with the outcome measures in the analysis to see if the carrier status influenced recovery.

### 2.10 Sample size

A recruitment target of 57 eligible participants was calculated such that the study would detect a difference of 20 mm^3^ in OBV with 90% power based on a 2:1 allocation ratio (active to placebo) and based on a standard deviation of 20 mm^3^ (Rombaux et al. 2012) with an assumed 10% drop-out rate accounting for the above MRI-based exclusions. This allowed greater capture of the number of adverse events in the intervention arm and ensured that the placebo arm contained 19 patients, an accepted minimum amount required for a feasibility or pilot study.

### 2.11 Harms

Definitions of harm based on the EU directive 2001/20/EC Article 2 were applied to the trial. All participants were contacted on a fortnightly basis to record and address any adverse events or adverse reactions. Unexpected serious adverse reactions and events were reported to the Research Ethics Committee and the sponsor.

### 2.12 Randomization

#### 2.12.1 Sequence generation

Participants were randomly assigned to either the intervention or the placebo group with a 2:1 allocation as per a computer-generated randomization schedule. The randomization was performed after the initial baseline MRI scan to ensure the eligibility criteria were fully met.

#### 2.12.2 Allocation concealment mechanism

The random allocation order was generated before the trial began and concealed from the research team by a CTU statistician. An interactive web randomization system, REDCap (Harris et al. 2009), was used to generate a “pack ID” for the dispensing trial product, for allocation of participants to one of the 2 groups, after the informed consent and baseline measures were completed. The method of using pack identification numbers ensured the blind was maintained, and medication could be dispensed by blinded members of the team.

#### 2.12.3 Implementation

Participants were allocated a participant number at the time of consent, with completion of baseline data in the case report form, enabling randomization. The treatment allocation was revealed and linked to that participant number.

#### 2.12.4 Blinding

All participants, care providers, and outcome assessors were blinded to the treatment allocation. No accidental or planned unblinding occurred. The data analysts were not fully blinded as they presented side-effect data by treatment group to the Safety Committee. required to know the group for the preparation of the DMEC reports.

### 2.13 Statistical methods

The comparison between groups was based on the intention-to-treat population.

#### 2.13.1 Olfactory bulb volume

For the primary outcome measure, changes in OBV were compared to the control group using a 2-sample test of outcome measures adjusted for baseline. The OBV was manually calculated by mapping voxels onto the T2 sequences of both olfactory bulbs (Rombaux et al. 2009). Two observers performed the calculations independently, and any volume discrepancy above 10% was assessed by a third observer to ensure matching. The second outcome measure of changes in orbitofrontal sulcus depth was analyzed in the same manner.

An adjusted analysis was conducted, adjusting for the corresponding baseline measures using a general linear model. A similar analysis to the OBV and sulcus measurement analysis was used for the TDI score from the Sniffin’ Sticks psychophysical smell test and the ODQ. We also assessed the correlation between the OBV measurements and the TDI scores using Pearson's correlation coefficient. A subgroup analysis to assess if the difference in outcomes between the groups is different for individuals with parosmia compared to those without was undertaken.

#### 2.13.2 Odor fMRI

Preprocessing (realignment, co-registration, segmentation, normalization, and smoothing) and subsequent statistical analyses were carried out using SPM12 (UCL, London, UK) (Penny et al. 2011). EPI images were realigned, co-registered to their anatomical image, normalized with the aid of each participant's individual segmented anatomical image, and smoothed with a 10 × 10 × 10 mm^3^ filter. Each GLM model consisted of 3 sessions of 128 volumes/participant/visit. The epoch regressor modeled odor as 10 s blocks with 15 s between blocks. There were 12 blocks/session. In addition, 6 movement regressors estimated in the realignment process during preprocessing were added to the model as regressors of no interest. Two general linear models (GLMs) per participant were estimated; 1 per visit. For each GLM, a contrast image, odor vs odorless air, was created for second-level analyses.

Before comparing the 2 groups, we investigated the overall brain activation pattern at visit 1 and visit 2, respectively, to ensure reliability. A 1-sample *t*-test was set up for each visit at a second level using the contrast images of odor vs odorless air. In addition, we added the TDI scores as covariates to investigate BOLD covarying with TDI scores. Subsequently, we set up a 2*2 factorial design with group (A and B) as one factor and time (visit 1 and visit 2) as another factor. Each participant's first-level contrast image (odor vs odorless air) from visit 1 was subtracted from the contrast image of visit 2 using the function ImCalc and added to a second-level summary 2-sample *t*-test (groups). In addition, we added a mean corrected covariate of TDI score change (visit 2 vs visit 1), for each level of the group factor, to investigate brain activation correlating with TDI scores. Difference scores for TDI were calculated both between the absolute TDI values and as a percent change. Results are reported as peak-statistics of regions of interest (ROIs)-corrected activations (*P* < 0.05) based on coordinates from Torske et al.—Table 1 “all odor” (Torske et al. 2022). Table 1 shows the ROIs that were used.

**Table 1:** Regions of interest for odor MRI.

Location	x (mm)	y (mm)	z (mm)
L Amygdala/Piriform cortex/Hippocampal gyrus	−22	0	−20
L Frontal pole	−26	32	−12
L Insula	−40	6	−10
L Orbitofrontal cortex	−34	22	−2
R Amygdala/Piriform cortex/Hippocampal gyrus	24	4	−18
R Insula/Frontal pole	38	6	−12
L Caudate	−12	8	2
R Insula/central opercular cortex	36	−6	12
Paracingulate gyrus anterior division	0	22	40

#### 2.13.3 Resting state fMRI

To understand the wider impact of anosmia on brain function, a resting state fMRI analysis was conducted with novel brain network and machine learning pipelines (Cusack et al. 2014). In a similar approach to Lee et al., who investigated olfactory functional networks in Parkinson's disease dementia and Alzheimer's dementia (Lee et al. 2017; Li et al. 2018), ROIs seed-based approach for the comparison of functional changes in the resting-state networks was used. The seed ROIs we aim to investigate are the olfactory bulb, olfactory tract, piriform cortex, and orbitofrontal cortex between groups. While OBV measurement has been widely used to investigate olfactory diseases, white matter integrity analyses are more recent. DTI analyses allow the evaluation of different fiber properties in white matter. Güllmar et al. showed that improvement of olfaction in chronic rhinosinusitis patients following surgery correlated with olfactory performance and DTI measures of cortical change (Güllmar et al. 2017).

### 2.14 Diffusion tensor imaging methods

In our study, the diffusion MRI analysis was conducted with a reproducible, robust, and efficient dMRI processing pipeline (Theaud et al. 2020). We used a diffusion tensor imaging sequence for olfactory bulb imaging and employed detailed microstructure profiling of the hippocampus, followed by global connectome-based machine learning to evaluate wider pathway effects. The use of advanced machine learning algorithms enabled us to go beyond simple correlations to make predictions of the effects of intervention (Breiman 2001; Alexander et al. 2007; Genicot et al. 2016).

#### 2.14.1 DTI processing and analysis

Structural T1-weighted images were processed with FreeSurfer recon-all, followed by FreeSurfer boundary-based co-registration of T1w to DWI. Diffusion data underwent preprocessing with MRtrix3, including denoising, Gibbs-ringing removal (degibbs), susceptibility-induced distortion correction, and bias-field correction. These steps reduced noise/artifacts and ensured accurate T1–DWI alignment (Fischl 2012; Tournier et al. 2019).

#### 2.14.2 Diffusion modeling and tractography

From the preprocessed DWIs, we derived tensor-based microstructure measures (e.g. apparent diffusion coefficient [ACD], fractional anisotropy [FrA], axial diffusivity [AD], and radial diffusivity [RD]). We estimated fiber orientation distributions using multi-shell, multi-tissue constrained spherical deconvolution, and generated anatomically constrained tractography. Streamlines were filtered with SIFT2 to improve biological plausibility (Tournier et al. 2019).

#### 2.14.3 Hippocampal microstructure analysis

Longitudinal hippocampus microstructure change was computed as session B − session A. Group differences (group A vs group B) in hippocampal ACD, FA, RD, and AD were assessed using unpaired, 2-sided *t*-tests. Metrics are reported separately for the left and right hippocampus.

#### 2.14.4 Connectome construction

We built SIFT-2 weighted subject-level connectivity matrices with anatomically constrained MRtrix3 Multi-Shell Multi-Tissue Constrained Spherical Deconvolution, generating 5 million streamlines. Standard FreeSurfer parcellation used the Desikan–Killiany cortical atlas alongside FreeSurfer volumetric subcortical labels in (34 left hemisphere, 34 right hemisphere cortical nodes, 16 subcortical nodes). Connectome matrices formed the basis for tractography-derived connectome analyses (Fischl 2012; Tournier et al. 2019).

#### 2.14.5 Connectome-based classification

Each subject's connectivity matrix and a design matrix of class labels were loaded for supervised learning. We first performed mass-univariate tests on all edges, thresholded by *P*-value, and extracted the largest connected component of significant edges as features (suprathreshold edge selection). Feature selection and model training were embedded within nested cross-validation. A linear discriminant analysis (LDA) classifier was trained, and edge-wise performance scores were aggregated to weight features (Serin et al. 2021).

## 3. Results

### 3.1 Participant flow

**Figure 1:**
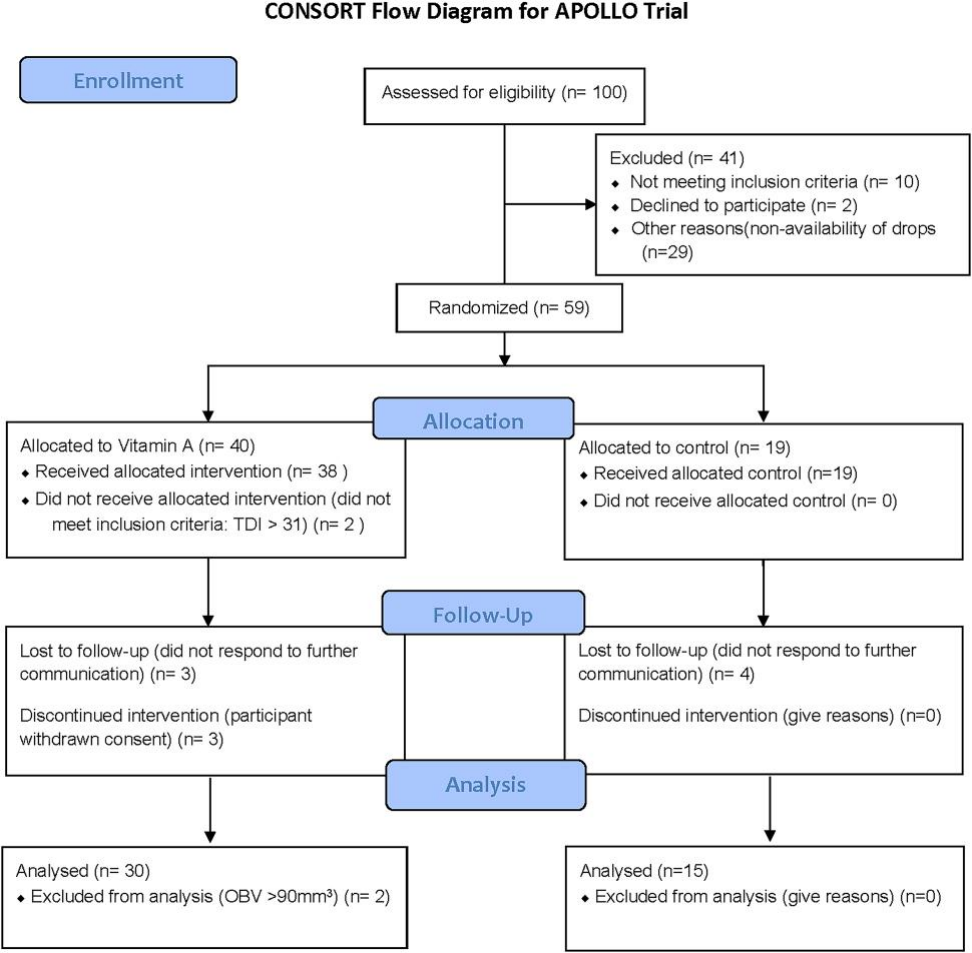
CONSORT flow diagram for APOLLO trial (Hopewell et al. 2025).

The APOLLO Trial was open for recruitment from 2022 July 01 to 2024 August 31, but closed from 2022 November 01 to 2024 January 31 due to a lack of vitamin A drop availability. The recruitment target for the trial was 57 participants, and all were recruited from the Norfolk Smell & Taste Clinic at the James Paget University Hospital. A total of 100 patients were assessed, with 57 meeting eligibility criteria and 47 participants attending their follow-up visits. The consort flow diagram is shown in Fig. 1.

### 3.2 Recruitment and baseline data

Eligible participants included 47 women and 10 men with a mean age of 47.2. All but one participant had experienced olfactory dysfunction due to COVID-19, but that one participant reported prior COVID-19 infection. Patient characteristics and baseline data are summarized in Table 2.

**Table 2:** Participant baseline characteristics.

Characteristics	Overall (*N* = 57)	Vitamin A (*N* = 38)	Placebo (*N* = 19)
Age at consent, mean (SD)	47.2 (12.41)	48.4 (12.21)	44.8 (12.80)
Sex at birth (%)			
Female	47 (82.5)	32 (84.2)	15 (78.9)
Male	10 (17.5)	6 (15.8)	4 (21.1)
Ethnicity (%)			
Asian—Other	1 (1.8)	1 (2.6)	0 (0.0)
Pakistani	1 (1.8)	1 (2.6)	0 (0.0)
White—British	54 (94.7)	35 (92.1)	19 (100.0)
White—Other	1 (1.8)	1 (2.6)	0 (0.0)
Has the participant had COVID-19? (%)			
Yes	56 (98.2)	37 (97.4)	19 (100.0)
No	1 (1.8)	1 (2.6)	0 (0.0)
Was the loss of smell caused by (%)			
COVID-19 infection	55 (96.5)	37 (97.4)	18 (94.7)
Other infection	2 (3.5)	1 (2.6)	1 (5.3)
Qualitative olfactory dysfunction (%)			
None	14 (24.6)	6 (15.8)	8 (42.1)
Parosmia only	15 (26.3)	13 (34.2)	2 (10.5)
Phantosmia only	5 (8.8)	4 (10.5)	1 (5.3)
Parosmia and phantosmia	23 (40.4)	15 (39.5)	8 (42.1)
OBV, mean (SD)	55.5 (16.76)	54.8 (18.96)	56.81 (11.88)
Right orbital sulcus depth, mean (SD)	7.96 (1.68)	7.91 (1.45)	8.07 (2.09)
Smell VAS mean (SD)	7.09 (2.28)	6.83 (2.27)	7.58 (2.27)
Taste VAS mean (SD)	5.71 (3.26)	5.03 (3.43)	7.00 (2.54)
ODQ score, mean (SD)	71.91 (15.18)	70.56 (15.93)	74.47 (13.67)
ODQ QoL subscore, mean (SD)	30.78 (11.29)	28.03 (10.60)	36 (10.97)
TDI, mean (SD)	22.07 (6.83)	22.52 (6.64)	21.16 (7.30)
TDI < 16, *n* (%)	13 (22.8%)	8 (21.1%)	5 (26.3%)
TDI 16 to 30.75 *n* (%)	44 (77.2%)	30 (79.0%)	14 (73.7%)
Hedonic direction, mean (SD)	−0.7 (0.85)	−0.75 (0.88)	−0.55 (0.80)
Hedonic range, mean (SD)	5.76 (1.94)	5.79 (2.01)	5.70 (1.83)
BDNF, mean (SD)	89.54 (31.24)	89.12 (28.55)	90.37 (37.11)

### 3.3 Numbers analyzed

After eligibility criteria were applied, 57 participants remained in the trial (38 received vitamin A and 19 received placebo), data from 30 participants in the vitamin A arm (A) and 15 in the placebo arm (B) were analyzed, with 2 participants excluded due to their combined OBV exceeding 90 mm^3^.

### 3.4 Outcomes and estimation

The mean increase in the combined OBV was 11.8 mm^3^ in the placebo group and 5.2 mm^3^ in the intervention group. Neither group achieved the 20 mm^3^ change set out in the power calculation. Apart from quality-of-life specific improvements in the ODQ score, there were also no significant changes in olfactory sulcus depth, TDI scores, and other domains of the ODQ scores, indicating that the groups did not appear to have had any notable improvements in outcome measures. The SSParoT scores showed differences in hedonic direction between the 2 groups, with the intervention group experiencing a more pleasant perception of odors at 12 wk compared to placebo, although this was not at the level of statistical significance. There was no meaningful difference in the hedonic range at 12 wk between the 2 groups. These findings are summarized in Table 3.

**Table 3:** Changes in primary and secondary outcome measures.

Outcome	Vitamin A group (SD) (*n* = 30)	Placebo group (SD) (*n* = 15)	Mean difference (95% CI)^a^	*P*-value^a^
Change in combined OBV	5.24 (13.08)	11.81 (9.20)	6.53 (−0.97, 14.03)	0.086
Change in right orbital sulcus depth	−0.52 (1.06)	−0.56 (1.21)	0.03 (−0.74, 0.68)	0.935
Reported change in smell	−0.70 (2.14)	−0.19 (1.76)	0.89 (−0.28, 2.05)	0.132
Reported change in taste	−0 (−3,0)	−0.5 (−2-0)	1.10 (−0.61, 2.81)	0.203
Change in ODQ score	−16.53 (19.54)	−9.67 (25.19)	10.93 (−2.69, 24.55)	0.113
Change in ODQ QoL subscore	−7.03 (9.73)	0.20 (11.97)	7.98 (1.91, 14.05)	0.011
Change in TDI score	2.85 (7.19)	3.88 (5.17)	0.40 (−3.27, 4.08)	0.825
TDI change > 5.5, *n* (%)	12/30 (40.0%)	6/15 (40.0%)	1.22^b^ (0.36, 4.20)	0.750
TDI ≥ 31	5/30 (16.7%)	4/15 (26.7%)	0.65^b^ (0.15, 2.84)	0.567
Change in hedonic direction	0.58 (0.94)	−0.06 (0.88)	−0.43 (−0.91, 0.04)	0.072
Change in hedonic range	0.50 (1.87)	0.56 (1.67)	−0.03 (−1.17, 1.11)	0.958
Change in BDNF	28.19 (32.91)	27.56 (29.28)	−0.06 (−18.41, 18.29)	0.995

^a^Adjusted for baseline values.

^b^Odds ratio.

#### 3.4.1 Olfactory fMRI

A total of 35 participants, group A (*n* = 24, mean age = 49.4 ± 9.9, 3 males) and group B (*n* = 11, mean age = 46.5 ± 13.5, 2 males), took part in both odor fMRI scanning sessions approximately 12 wk apart.

#### 3.4.2 Brain activation odor vs odorless air visit 1

**Figure 2:**
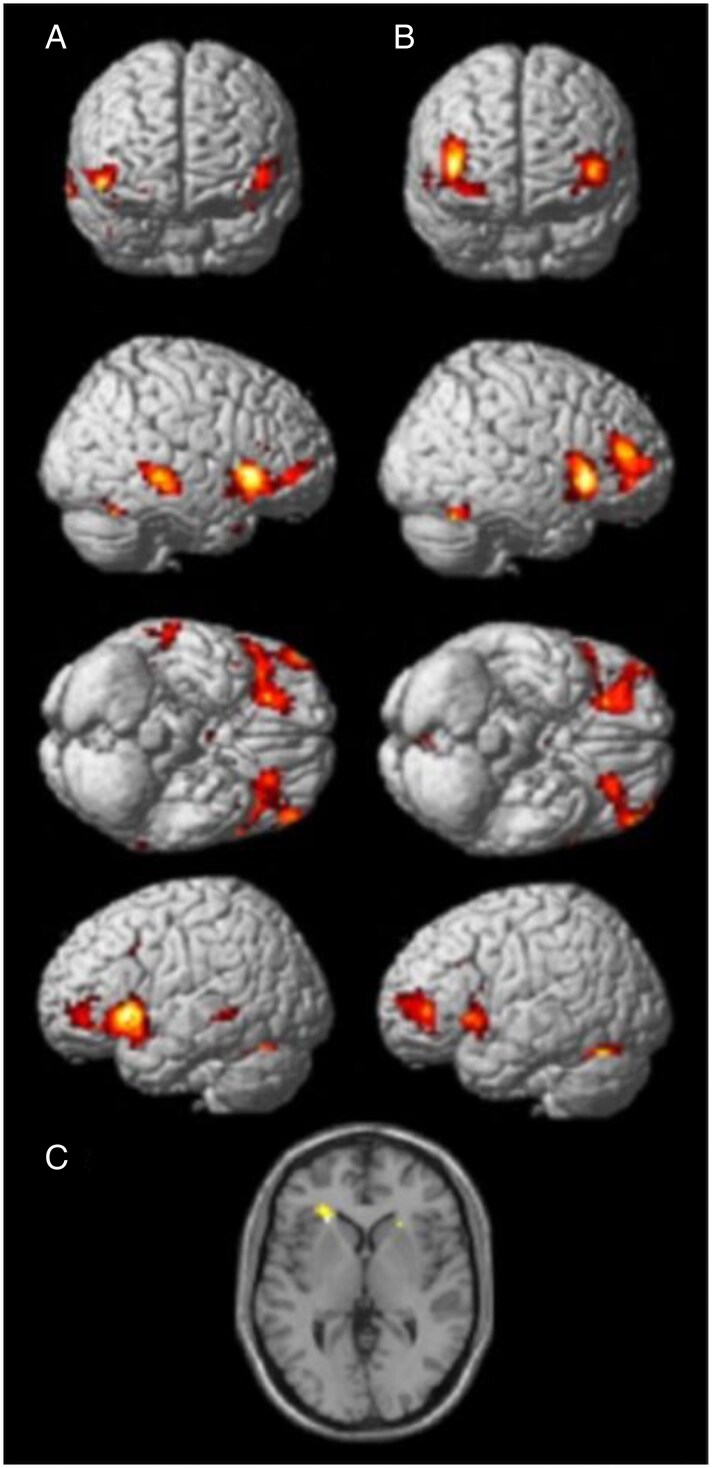
Overall fMRI olfactory activation.

Significant activations were found in typical olfactory brain areas when participants received odors during fMRI scanning when compared to odorless air. As outlined in Table 4 and Fig. 2, activations were found bilaterally in the posterior parts of orbitofrontal cortex, amygdala, piriform cortex, and insula cortex.

**Table 4:** Significant brain activations when participants perceive odors compared with odorless air at visit 1 and visit 2.

Location	*P* ROI (FWE)	*T*	*P* peak (uncorr)	X (mm)	Y (mm)	Z (mm)	ROI coordinate	ROI size (radius, mm)
Visit 1								
L Orbitofrontal cortex	0.04	3.66	0.00	−45	17	−1	−34 22 −2	10
L Posterior orbital cortex/Insula	0.01	3.92	0.00	−24	32	−7	−27 32 −13	10
L Amygdala/Piriform cortex/Insula	0.00	4.37	0.00	−30	8	−13	−22 0 −20	20
R Orbitofrontal cortex	0.00	4.72	0.00	21	32	−10	24 32 −16	10
R Amygdala/Piriform cortex/Insula	0.00	4.71	0.00	30	5	−13	24 4 −18	10
Visit 2								
L Piriform cortex	0.021	2.98	0.00	−36	5	−13	−40 6 −10	5
L Orbitofrontal cortex	0.04	3.32	0.00	−27	35	−7	−34 22 −2	10
L Posterior orbital cortex/Insula	0.003	4.16	0.00	−24	32	−10	−27 32 −13	10
R Piriform cortex	0.05	3.2	0.00	36	8	−13	38 6 −12	10
R Orbitofrontal cortex	0.001	4.35	0.00	24	35	−10	24 32 −16	10
R Amygdala/Piriform cortex/Insula	0.03	2.96	0.002	33	5	−16	24 4 −18	10

FWE, family wise error corrected *P*-value; uncorr, uncorrected *P*-value; x, y, z, peak location in MNI space.

#### 3.4.3 Brain activation odor vs odorless air visit 2

Significant activations were found in typical olfactory brain areas when participants received odors during fMRI scanning when compared to odorless air during the second visit. As outlined in Table 4 and Fig. 2, activations were found bilaterally in the posterior parts of orbitofrontal cortex, piriform cortex, and insula cortex.

#### 3.4.4 Brain activation covarying with TDI scores

No brain activation was found to correlate significantly with TDI scores at the first visit (*P* > 0.05, ROI). Brain activation correlated significantly with TDI scores at visit 2 in the left anterior insula/posterior orbitofrontal cortex (−27 32 −13; *t*(33) = 3.46, *P* = 0.026) and left orbitofrontal cortex (−34 22 −2; *t*(33) = 4.71, *P* < 0.001) (Fig. 2).

#### 3.4.5 Group–time comparisons

We found no significant interaction between group and time, neither when comparing contrasts nor when comparing BOLD correlating with TDI scores (*P* > 0.05, ROI). We found no significant main effect of group, neither when comparing contrasts nor when comparing BOLD correlating with TDI scores (*P* > 0.05, ROI). We found no significant main effect of time when comparing contrast (*P* > 0.05, ROI).

No brain activation change was found to correlate significantly with TDI score changes between the 2 visits (*P* > 0.05, ROI), across participants, neither for absolute differences in TDI scores nor percent change (*P* > 0.05, ROI).

#### 3.4.6 Ancillary analyses

No brain activation change was found to correlate significantly with TDI score changes between the 2 visits (*P* < 0.05, ROI). We note a significant trend of *F*(1,32) = 14.8, *P* = 0.16 in right insula (39 23 −6). We found no significant main effect of group (*P* < 0.05, ROI). We note that the left posterior orbital cortex and the left insula display a potential significant trend (Table 4, Fig. 2).

#### 3.4.7 Diffusion tensor imaging results

The analysis of longitudinal changes in hippocampal microstructure revealed no significant differences between the intervention and control groups. In the left hippocampus, microstructure change scores were not significant for ADC (*t* = −0.65, *P* = 0.526), FA (*t* = 1.08, *P* = 0.295), RD (*t* = −1.00, *P* = 0.329), or AD (*t* = 0.07, *P* = 0.944). Similarly, the right hippocampus showed no significant group differences for ADC (*t* = 0.61, *P* = 0.550), FA (*t* = 1.62, *P* = 0.123), RD (*t* = 0.38, *P* = 0.707), or AD (*t* = 0.95, *P* = 0.355).

Connectome-based machine learning was also unable to accurately classify subjects group membership. The LDA classifier performed at a level comparable to chance, with a cross-sectional classification accuracy of 56.3% for session A and 51.1% for session B. These performance metrics indicate that the model could not observe differential patterns in participant structural connectomes after intervention.

#### 3.4.8 ApoE gene status

Samples were collected from 30 participants. The majority of participants had ApoE 3/3 alleles. When comparing intervention and control groups, the average improvement in TDI score and OBV after 12 wk was higher in most of the ApoE groups in the intervention arm (Table 5).

**Table 5:** ApoE results and outcomes.

	Baseline TDI mean (SD)	12 wk TDI mean (SD)	Average TDI change	Baseline combined OBV mean (SD)	12 wk combined OBV mean (SD)	Average OBV change
**Vitamin A group**						
2;3 (*n* = 1)	18.00	29.25	11.25	46.67	54.71	+8.04
3;3 (*n* = 8)	24.47 (4.74)	28.26 (5.98)	3.79	61.12 (13.64)	74.55 (10.33)	+13.43
3;4 (*n* = 4)	20.63 (8.48)	25.69 (7.60)	5.06	58.11 (8.60)	69.91 (19.01)	+11.80
**Placebo group**						
2;3 (*n* = 1)	27.50	26.75	−0.75	72.11	73.30	+1.19
2;4 (*n* = 1)	30.00	22.00	−8.00	88.73	122.48	+33.75
3;3 (*n* = 13)	22.40 (5.90)	24.93 (6.08)	2.53	54.77 (17.22)	65.52 (17.00)	+10.75
3;4 (*n* = 2)	25.90 (2.65)	29.25 (3.89)	3.38	66.38 (3.64)	76.86 (3.36)	+10.48

Due to limited numbers, statistical analysis was limited to the ApoE3/3 intervention and control groups. In the ApoE3/3 intervention group, the average participant age was 42 years and 88% were females, whereas in the ApoE3/3 control group, the average age was 48 years with 84.6% being female. The mean difference in change in TDI scores and OBV between intervention and control groups was calculated using an independent samples *t*-test. The mean difference in the change in TDI scores between the groups was −0.21 [95% CI: −8.57 to 8.15], and the result was not statistically significant, *t*(17)=−0.058, *P* = 0.958. The mean difference in OBV change between the intervention and control groups was 6.16 [95% CI: −5.47 to 17.79], which highlights that the intervention group had a greater increase. However, this difference was not statistically significant (*t*(14) = 1.176, *P* = 0.259).

### 3.5 Harms

Twenty-six participants experienced minor side effects from the trial medication, primarily nasal obstruction and discomfort. One participant had epistaxis during the trial period, which was reviewed at the local ear, nose, and throat (ENT) department for nasal cautery. One participant experienced symptoms of vitamin A/retinoid toxicity, including peeling and itchy skin and dry and cracked lips. Further blood testing did not show elevated serum vitamin A levels, nor derangement of liver function, and the participant was recommenced on the trial medication after dose reduction.

## 4. Discussion

### 4.1 Key results

This mechanistic study has not demonstrated that vitamin A drops are beneficial in treating COVID-19-related PIOD. Neither the primary nor any of the secondary objective and subjective outcome measures showed any statistically or clinically significant differences between the 2 arms of the trial, except a small quality-of-life improvement in a subsection of the ODQ. Notably, 9 (20%) participants were normosmic by the follow-up visit, regardless of the intervention (4 vitamin A, 5 placebo), indicating spontaneous recovery. It was also evident that 18 (40%) participants improved their sense of smell by the MCID of ≥ 5.5 points on the TDI score (6 placebo, 12 vitamin A), indicating the rate of natural recovery of olfactory function for COVID-19-related PIOD.

### 4.2 Interpretation

The evidence on which the rationale for the study was founded is described in the introduction. Since trial commencement, 2 additional studies have published their findings. A pilot study conducted in China compared a short course of oral vitamin A and aerosolized diffuser olfactory training (OT) with OT alone and no intervention for patients with COVID-19-related PIOD (Chung et al. 2023). The sample size was very small (*n* = 24), with only 10 participants in the combination therapy group and yet the authors claimed to demonstrate a positive benefit from the combination treatment. A larger RCT conducted in Iran also used oral vitamin A as part of a 3-arm design, including in combination with OT and OT alone, but merely demonstrated the benefit of OT over no intervention, as there was no clear difference in olfactory performance between the 2 intervention arms (Taheri et al. 2024). In both studies, the vitamin A was taken systemically, going against prior evidence of any benefit by oral ingestion (Duncan and Briggs 1962; Reden et al. 2012). Both studies did, however, also include a cohort of COVID-19-related PIOD and as such are a comparable population sample. The pre-pandemic studies included mixed viral/infective etiologies and so may not represent comparable populations based on the pathophysiology described above. No other studies have subjected the use of topical vitamin A to investigation using a mechanistic approach such as the one undertaken in the APOLLO trial, either in a COVID-19-specific population or other non-COVID cases of PIOD.

The APOLLO trial was conducted with vitamin A drops self-administered by participants via the Kaiteki position to maximize distribution to the olfactory cleft (Mori et al. 2016). Other novel application methods, such as mucoadhesive agents, hydrogels, and nanocarriers, have been explored for drug delivery into the olfactory cleft (Bourganis et al. 2018; Gänger and Schindowski 2018). These may potentially be suitable for the delivery of the fat-soluble esters of vitamin A to the olfactory cleft and are worthy of consideration in future studies. However, their likely need for direct administration under endoscopic guidance presents a significant challenge to implementation in trial settings (Espehana et al. 2023).

In terms of the ApoE gene status, the participants with ApoE2/3 alleles in the intervention group have achieved the largest improvement in TDI scores, in line with prior research, which shows this to be neuroprotective (Josefsson et al. 2017; Olofsson et al. 2020). However, no ApoE 4/4 participants, who have the greatest decline in olfaction and risk for Alzheimer's dementia, were identified in our study (Ward et al. 2012). In conjunction with the very limited sample size, this study has been unable to demonstrate any relationship between the ApoE gene status and response to vitamin A. Significantly larger populations will be required with larger cohorts of ApoE4/4 participants to further assess the impact of the ApoE gene on olfactory function in future studies. Similarly, a focus on ApoE2 in future olfactory trials may well be important given its neuroregenerative relevance.

### 4.3 Limitations

All of the recruited participants had COVID-19-related PIOD, except for one who reported the timing of their COVID-19 infection separate to the onset of their PIOD; it is deemed likely this case was also COVID-19-related. This, therefore, limits the study findings' applicability to other infective causes of PIOD. At the point of conception of the trial, it was anticipated that the study would recruit a mixture of viral etiologies typically seen in the community, but the population presenting after recruitment opened was almost exclusively COVID-19-related.

We did not collect a full dataset for all outcome measures on all 55 participants in the trial due to multiple factors, including the long scan sequences, claustrophobia, lack of wider bore magnet to accommodate larger BMI participants comfortably for long time, geographical distance of the participant to make a timely follow-up scan visit, and technical difficulties with the olfactometer.

### 4.4 Generalizability

Our results do not support the routine use of vitamin A drops for patients with COVID-19-related PIOD. Although a limited change of quality of life was present, we did not see convincing evidence of a group effect, suggesting vitamin A should be considered for a further RCT in this population of COVID-19-related PIOD. The mechanism for injury to the olfactory system in COVID-19 is different from that seen in other viruses, with robust evidence pointing to sustentacular cells as the primary target cell type for COVID-19. ACE2 and TMPRSS2 expression is raised in sustentacular cells, with their infection and subsequent inflammatory signaling demonstrated in postmortem studies (Khan et al. 2021; Finlay et al. 2022). Furthermore, the Omicron variant of COVID-19 was associated with relatively low levels of olfactory dysfunction, coinciding with its impaired ability to utilize the TMPRSS2 pathway in comparison to previous variants (Chen and Wang 2023). As such, the regenerative effects of vitamin A on ORNs may not be sufficient in counteracting COVID-19 infection of sustentacular cells and its subsequent downstream disruption of ORN function (Rawson and LaMantia 2006; Verma et al. 2022). Further study on non-COVID PIOD-affected patients may be a consideration given the differing mechanisms of action of viral pathophysiology, but other therapeutic agents should be explored for COVID-related PIOD. The study has also enabled the research team to develop a robust method of delivering and analyzing such studies through the University of East Anglia Brain Imaging Centre and anticipate being well placed to conduct further such proof-of-concept studies, subject to further funding. The need for effective treatments remains ever present given the scale of PIOD and other olfactory disorders and their impact (Garden et al. 2023; Whitcroft et al. 2023; Glibbery et al. 2024; Lee et al. 2024). Our findings of spontaneous recovery in 1 in 3 participants are in keeping with other recent work on recovery of olfactory function after COVID-19 (van Dijk et al. 2025) and also with historic recovery rates for PIOD pre-COVID-19 (Reden et al. 2006).

## 5. Other information

Public and patient involvement was provided by Jim Boardman on behalf of SmellTaste (www.smelltaste.org.uk).

### 5.1 Protocol

The published protocol can be found online at: https://rdcu.be/elYva (Kumaresan et al. 2023).

## Data Availability

Participant data are stored at the central database on servers based at the Norwich Clinical Trials Unit (NCTU). This is accessible only to members of the APOLLO trial team at NCTU, and external regulators and reviewers if requested. The servers are protected by firewalls and are patched and maintained according to best practices. The physical location of the servers is protected physically and environmentally in accordance with the University of East Anglia's General Information Security Policy 3 (GISP3: Physical and environmental security). Following completion of the trial, the database is retained on the servers of NCTU for ongoing analysis and data query requests. The identification, screening, and enrollment logs, linking participant identifiable data to the pseudo-anonymized PID, will be held locally by the trial site. This will either be held in written form in a locked filing cabinet or electronically in password-protected form on hospital computers. After completion of the trial, the identification, screening, and enrollment logs will be stored securely by the sites for 5 years unless otherwise advised by NCTU.
